# Neoadjuvant toripalimab combined with gemcitabine and cisplatin in resectable locally advanced head and neck squamous cell carcinoma (NeoTGP01): An open label, single-arm, phase Ib clinical trial

**DOI:** 10.1186/s13046-022-02510-2

**Published:** 2022-10-12

**Authors:** Xiaotao Huang, Qiaodan Liu, Guihua Zhong, Yingpeng Peng, Ye Liu, Lizhong Liang, Haiyu Hong, Weineng Feng, Shuang Yang, Yaqin Zhang, Shiping Xian, Zhanyu Li, Yuling Zhou, Zhaoyuan Zhang, Wen Jiang, Jun Liang, Zhi-gang Liu

**Affiliations:** 1 The Cancer Center of the Fifth Affiliated Hospital of Sun Yat-Sen University, Zhuhai, 519000 Guangdong China; 2 Guangdong Provincial Key Laboratory of Biomedical Imaging, Fifth Affiliated Hospital of Sun Yat-Sen University, Zhuhai, Guangdong China; 3 Department of Pathology, Fifth Affiliated Hospital of Sun Yat-Sen University, Zhuhai, Guangdong China; 4 Department of Oral and Maxillofacial Surgery, Fifth Affiliated Hospital of Sun Yat-Sen University, Zhuhai, Guangdong China; 5 Department of Otolaryngology, Fifth Affiliated Hospital of Sun Yat-Sen University, Zhuhai, Guangdong China; 6 Head and Neck/Thoracic Medical Oncology, Foshan First People’s Hospital, Foshan, Guangdong China; 7 Department of Radiology, Fifth Affiliated Hospital of Sun Yat-Sen University, Zhuhai, Guangdong China; 8 Department of Radiation Oncology, The University of Texas MD Anderson Cancer Center, Houston, TX USA; 9 Department of Stomatology of the Seventh Affiliated Hospital of Sun Yat-Sen University, Shenzhen, 518000 Guangdong China; 10 Cancer Center, Dongguan People’s Hospital (Affiliated Dongguan Hospital to Southern Medical University), Dongguan, Guangdong China

**Keywords:** Head and neck squamous cell carcinoma, Immunotherapy, Neoadjuvant treatment, Pathological response rate

## Abstract

**Background:**

Neoadjuvant programmed death receptor-1 (PD-1) inhibitors have drawn increasing attention in locally advanced head and neck squamous cell carcinoma (HNSCC). In this study, we investigated the safety and efficacy of gemcitabine and cisplatin (GP), combined with a PD-1 inhibitor, in patients with locally advanced HNSCC.

**Materials and methods:**

A total of 23 eligible patients were administered two cycles of toripalimab and GP followed by surgical resection. The primary endpoints were safety, treatment-related adverse events (TRAEs), and non-operation delay rates. The secondary endpoints consisted of pathological complete response (pCR) rate, major pathological response (MPR) rate, objective response rate (ORR), and R0 resection rate.

**Results:**

The incidence of TRAEs from grades 1 to 4 was 43.5%, 34.8%, 13.0%, and 8.7%, respectively. Grade 3/4 TRAEs included neutropenia, fatigue, hyperglycemia, nausea and vomiting, decreased appetite, rash, and diarrhea. No treatment-related surgical delay was observed. The radiographic response rates were 5.0% (CR), 40.0% (PR), and 55.0% (SD). The ORR reached 45.0%. Eighteen patients underwent successful surgical resection. The R0 resection rate was 100%. The pathological response rates were 16.7% (pCR), 27.8% (MPR, two of five near-pCR), 16.7% (PPR), and 38.8% (NPR). CD4, CD8, CD20, and CD38 expression in the tumors significantly increased after neoadjuvant chemotherapy. The increase in CD20 levels after neoadjuvant treatment in patients with pCR/MPR was significantly higher than in patients with PPR/NPR.

**Conclusion:**

Triweekly neoadjuvant toripalimab-GP is feasible and achieves promising pCR and MPR rates in patients with resectable locally advanced HNSCC.

**Trial registration:**

Chinese clinical trial registry, ChiCTR2100043743, Registered 27 Febrary 2021- Retrospectively registered, http://www.chictr.org.cn/showproj.aspx?proj=120570

**Supplementary Information:**

The online version contains supplementary material available at 10.1186/s13046-022-02510-2.

## Introduction

Head and neck squamous cell carcinoma (HNSCC) is the eighth most common cancer worldwide, accounting for 4.6% of all cancer cases, with 744,994 new cases and 363,339 deaths worldwide in 2020 [1]. Approximately 60–70% of patients present with locally advanced stage or distant metastasis at the time of diagnosis [2]. Patients with potentially resectable locally advanced HNSCC have up to a 50% risk of local recurrence and distant metastasis within three years after regional resection and adjuvant radiotherapy or chemoradiotherapy [3]. The 5-year survival rates of patients with stage III and IVA are 61 and 32%, respectively, despite multidisciplinary and multimodal treatments [4].

Among patients with HNSCC, programmed death receptor-1 (PD-1) inhibitors were first tested in a population with recurrence/metastasis [5, 6] and gave encouraging results. Neoadjuvant immunotherapy has attracted significant attention in recent years because of its potential to elicit heightened antitumor immune responses in the presence of tumor tissues that act as a source of neoantigens. The preliminary results of trials that used neoadjuvant immunotherapy with a PD-1 inhibitor alone in patients with locally advanced resectable HNSCC have recently been reported [7, 8]; these trials have confirmed the safety of the PD-1 inhibitor, demonstrating that 33–44.4% of patients achieved a pathological response. A 2-year postoperative follow-up showed that no locoregional relapse or distant metastasis occurred in these patients [8]. Compared with the PD-1 inhibitor alone, the combination of a PD-1 inhibitor and chemotherapy improved the major pathological response (MPR) up to 85.7% from no more than 60% in treating non-small cell lung cancer (NSCLC). Long-term follow-up data demonstrated that patients with a superior pathological response had a longer disease progression survival (DFS) and overall survival (OS) [9]. Considering NSCLC as a precedent, further breakthroughs may be possible using neoadjuvant chemoimmunotherapy for treating HNSCC.

Studies in which a gemcitabine and cisplatin (GP) regimen has been applied in HNSCC [10, 11] have demonstrated promising efficacy and a favorable toxicity profile. Nasopharyngeal carcinoma (NPC) is the preferred standard regimen for induction chemotherapy and is recommended by several guidelines [12]. The GP regimen has shown better efficacy and a lower incidence of ≥ 3 treatment-related adverse events (TRAEs) than the PF regimen (cisplatin and fluorouracil) in NPC [13]. Although there is no direct comparison of the efficacy between GP and TPF (cisplatin, fluorouracil, and docetaxel) in HNSCC, GP was estimated to be more cost-effective than TPF in China [14]. In addition, gemcitabine reportedly promotes PD-L1 expression and increases the ratio of CD8^+^ and CD4^+^ T cells, resulting in a synergistic antitumor effect with anti-PD-1 therapy [15].

Toripalimab, a PD-1 inhibitor, has been approved for the treatment of NPC, esophageal carcinoma, melanoma, and soft tissue sarcoma in China [16]. Here, we report the results of a phase Ib clinical trial that investigated the safety and efficacy of neoadjuvant toripalimab combined with GP in resectable locally advanced HNSCC.

## Methods and materials

This trial was designed as an open-label, single-arm, phase Ib clinical trial. A total of 23 eligible patients were enrolled successfully from January 2021 to January 2022. This trial was both registered at ClinicalTrials.gov and Chinese Clinical Trial Registry, respectively numbered NCT04947241 and ChiCTR2100043743.

### Patients

The inclusion criteria were patients aged 18 to 70 years, with histologically defined HNSCC, and staged III-IVB based on the American Joint Committee on Cancer, eighth edition (AJCC 8.0). In addition, experienced surgeons and oncologists assessed the participants to validate the potential of radical surgical resection. Other inclusion criteria were an expected survival of more than 6 months, an Eastern Cooperative Oncology Group performance status score of 0 to 2, and normal organ function. The primary exclusion criteria were allergy to any study drug, bi-primary carcinoma, immune deficiency, and active hepatitis. The complete criteria are listed in the Supplementary Data. Informed consent was obtained from all the study participants.

### Study design

Patients who met the criteria were administered two cycles of intravenous toripalimab (at a dose of 240 mg on day 1), gemcitabine (at a dose of 1000 mg/m^2^, days 1 and 8), and cisplatin (at a dose of 80 mg/m^2^, day 1); 21 days were set as a cycle. Radical surgical resection was planned for approximately four weeks after the first day of the second treatment cycle. The surgical project proceeded according to the standard international guideline with baseline evaluation; little modification of the extent of dissection was accepted, although tumor regression was observed after neoadjuvant therapy. Postoperative adjuvant therapy or observation was decided by investigators, mainly depending on the initial tumor stage, radiographic and pathologic response to neoadjuvant treatment, surgical resection margin, and lymph node pathologic positive rate in accordance with the standard treatment guidelines. For patients with T3/4 and N2/3 stages, positive surgical resection margins, or positive lymph nodes, adjuvant radiotherapy was suggested as a supplement [17].

For enrollment, all candidates underwent the following preexamination: pre-treatment biopsy, magnetic resonance imaging (MRI), positron emission tomography-computed tomography (or computed tomography), hepatic and renal function tests, and coagulation function test. MRIs were scheduled at three time points: pre-treatment, pre-surgery, and post-surgery, while the tissue samples were obtained by biopsy and surgery.

This research consisted of two phases: safety run-in and case development. In the first phase, we initially observed six patients for 90 days from the first day of their treatment (or until 30 days after surgery) to evaluate whether dose-limited toxicity (DLT) occurs in two or more of them. DLT was explained at length as follows: (a) Grade 3 toxicity, lasting for more than seven consecutive days, and (b) Grade 4 toxicity, excluding asymptomatic blood and biochemical abnormalities. If DLT were recorded in no more than one patient, the trial would continue until the scheduled size of the population was met; otherwise, the trial would stop. Further investigation would proceed to review the data thoroughly and modify the protocol.

### Outcome

The primary endpoint was safety, including treatment-related adverse events (TRAEs) and non-surgical delay rate. The adverse events in all patients were observed and recorded based on the National Cancer Institute Common Terminology Criteria for Adverse Events, version 5.0 (CTCAE 5.0), 90 days after the first day of treatment (or 30 days after surgery). The investigators determined whether the AEs were related to the treatment according to the study protocol and regulatory requirements. The TRAEs were screened and included in the statistical analyses. The surgical delay was defined as the date of radical surgery performed due to TRAEs exceeding the planned date by more than 4 weeks. The secondary endpoints consisted of the pCR rate, MPR rate, ORR, and R0 resection rate for patients who had completed resection. Furthermore, exploratory endpoints contained latent immunological responses in the blood and tumor samples.

### Radiological and pathological response assessment

Radiographic responses to neoadjuvant therapy were evaluated by radiologists according to RECIST 1.1. The pathological response was defined as the percentage of residual viable tumor (RVT) in the total tumor bed in the primary tumor site by hematoxylin and eosin staining and immunohistochemistry, assessed by three experienced pathologists; the evaluation of any individual was independent of each other. No RVT was evaluated as pCR, whereas MPR, partial pathologic response (PPR), and no pathologic response (NPR) were graded according to the percentage of RVT: 0 < RVT ≤ 10%, 10% < RVT ≤ 50%, and RVT > 50%.

### Immunohistochemistry

Immunohistochemistry was performed on paraffin slices derived from tumor tissues before and after neoadjuvant chemoimmunotherapy. Whole sections were stained for PD-L1, CD4, CD8, CD20, CD68, and CD38 and scanned using a Kscanner (KFBIO, Ningbo Kongfong Biotech International). Except for PD-L1, the remaining biomarkers were quantified in two dimensions: intensity and area. After examining all the scanned slices, two professionally trained pathologists analyzed and agreed on the standard for six intensity grades from 0 to 5 for each biomarker within all positive cells. Meanwhile, six regions of interest (ROI) of each stained slice were selected randomly in the identified range of the tumor bed under a 200 × magnified visual field. The immunohistochemical score of each ROI was calculated using the following algorithm: ∑intensity grade × corresponding area%. Specifically, when multiple intensity grades occurred in the same ROI, the expression score was calculated by summing them [18]. Regarding PD-L1, the combined positive score (CPS), as described previously, was used to quantify the expression of tumor cells and tumor-associated immune cells [19, 20]. Meanwhile, we analyzed the percentage of positive PD-L1 cells in the total tumor cells for pre- and post-treatment specimens. The assessments were performed by two independent pathologists.

### Statistical analysis

This was a single-arm prospective clinical trial without normative sample size calculations. The enrollment of 20 to 30 subjects followed the typical sample size of phase Ib exploratory studies [21]. The study recruited subjects continuously after successfully going through the safety run-in phase of the initial 6 patients until the sample size was satisfied. Side effects and AEs were monitored and recorded rigorously during the observation period for descriptive statistics. Statistical analyses consisted of an independent-samples T test, paired-samples t-tests, and non-parametric tests. For treating the data of pre- and post- treatment from the same individuals, we conducted paired-samples t-tests to make the analyses. In univariate analyses of different populations, independent sample T test was adopted for data that meet both normality and homogeneity of variance, otherwise non-parametric test was adopted. The correlation between any immunohistochemical index and pathological response was estimated using Spearman’s rank correlation coefficient. *P* values were two-sided, and the significance level was 0.05. Statistical analyses were conducted using SPSS and R software packages.

### Funding

This project was partly funded by an investigator-initiated trial at the hospital. Toripalimab was kindly provided by Shanghai Junshi Biosciences Co., Ltd.

## Results

### Patients

**Fig. 1:**
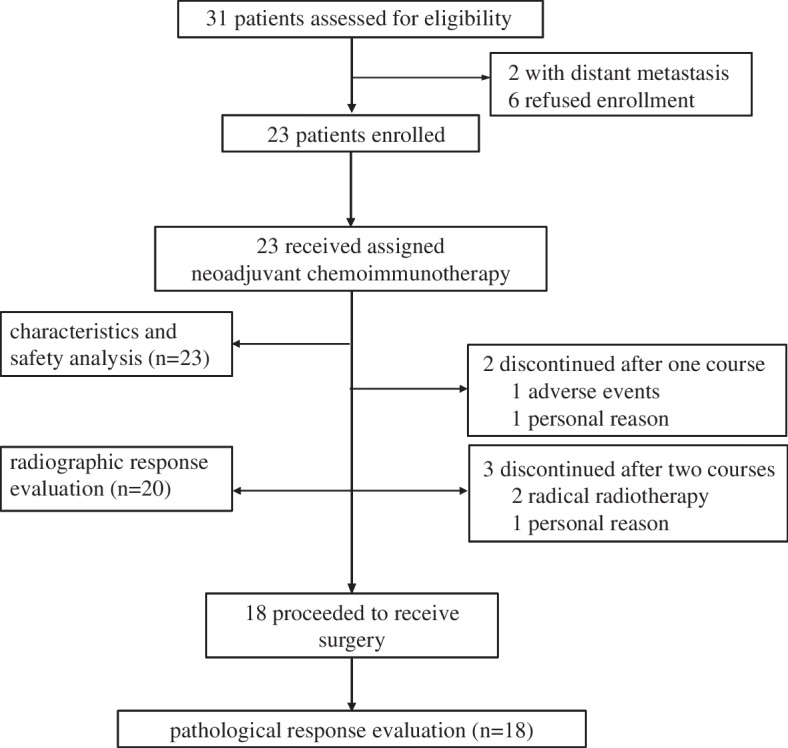
Clinical trial flow diagram

Between January 2021 and January 2022, 31 candidates were completed the examination before enrollment and 23 eligible patients were enrolled in this trial (Fig. 1). Their baseline clinical information is presented in detail in Table 1. All enrolled patients had newly diagnosed HNSCC, stage III to IVB, according to the American Joint Committee on Cancer (AJCC 8^th^) staging system. The primary lesions were predominantly located in the oral cavity (16/23, 69.7%). Of the other cases, three (13.0%) were of the larynx, three (13.0%) were of the oropharynx, and one (4.3%) was of the hypopharynx. More than half (52.1%) of the patients had stage T4 disease. The lymph nodes were positive in 18 patients (78.3%). The majority of the patients were HPV-negative (the percentage of p16 + cells was < 75%) (17/23, 73.9%).

**Table 1:** Patient characteristics

Variables	Patients (*N* = 23)	%
Age at enrollment (years), median (range)	56 (31–70)	
Sex		
Male	21	91.3
Female	2	8.7
Tumor Site		
Oropharynx	3	13.0
Root of tongue	1	4.3
Tonsil	2	8.7
Larynx	3	13.0
Hypopharynx	1	4.3
Oral Cavity	16	69.7
Tongue	11	47.8
Oral floor	2	8.7
Gingiva	1	4.3
Cheek	1	4.3
Mandible	1	4.3
T category		
T1	1	4.3
T2	2	8.7
T3	8	34.8
T4a	11	47.8
T4b	1	4.3
N category		
N0	5	21.7
N1	5	21.7
N2	8	34.8
N3	5	21.7
AJCC stage (the eighth edition)		
III	8	34.8
IVA	12	52.2
IVB	3	13.0
Smoking		
Never smoking	2	8.7
No smoking for 2 years	3	13.0
Smoking	18	78.3
HPV status (p16 expression)		
< 25%	14	60.9
26%–75%	3	13.0
> 75%	4	17.4
NA	2	8.7
PD-L1 expression CPS		
< 1	1	4.3
1 ~ 19	8	34.7
≥ 20	12	52.2
NA	2	8.7

*Abbreviations*: *AJCC* American Joint Committee on Cancer, *HPV* Human papillomavirus, *PD-L1* Programmed death ligand-1, *CPS* Combined positive score

### Treatment characteristics

Of the total 23 patients, 18 (78.3%) patients who completed the two cycles of neoadjuvant treatment underwent radical surgical resection at an average of 33 days after the first day of the second cycle of administration (range: 22–63 days). The other five (21.7%) participants failed to complete the full course of treatment as planned and withdrew from the study; two patients were administered only one cycle of neoadjuvant treatment due to toxicities and personal reasons; three patients completed two cycles followed by definitive radiotherapy instead of surgery, with the goal of organ preservation due to tumor regression after two courses of toripalimab plus GP.

### Safety

A total of 23 patients were included to evaluate the safety of neoadjuvant toripalimab-GP. TRAEs are summarized in Table 2. We passed the safety run-in phase with no more than one case of TRAE recorded in the first six subjects. No treatment-related deaths occurred during this period. The incidence of TRAEs from grades 1 to 4 was 43.5, 34.8, 13.0, and 8.7%, respectively. The most common TRAEs were decreased appetite and anorexia (39.1%), asthenia and fatigue (26.0%), nausea (26.0%), and vomiting (26.0%). Only two subjects (8.7%) experienced transient grade 4 TRAEs, of which one developed diabetic ketoacidosis after radical surgery and resolved within a week with active insulin and supporting treatment. The other patient experienced serious TRAEs, including febrile neutropenia, diarrhea, and vomiting after one course of treatment. Grade 4 TRAEs lasted for approximately four days, and remission and complete recovery were achieved after symptomatic treatment for nearly half a month. The median interval from the first day of the second administration to the date of surgical resection was 33.5 days. No treatment-related surgical delay was observed. The mean length of surgery-related hospital stay was 15.3 days, with a maximum of 21 days and a minimum of 6 days.

**Table 2:** Treatment-related adverse events (TRAEs) identified by investigators

	**Grade 1**		**Grade 2**		**Grade 3**		**Grade 4**	
	No	%	No	%	No	%	No	%
All patients with an event	10	43.5	8	34.8	3	13.0	2	8.7%
Asthenia or fatigue	4	17.4	1	4.3	1	4.3	0	0.0
Nausea	2	8.7	3	13.0	1	4.3	0	0.0
Vomiting	2	8.7	3	13.0	1	4.3	0	0.0
Decreased appetite or anorexia	6	26.1	2	8.7	1	4.3	0	0.0
Constipation	1	4.3	2	8.7	0	0.0	0	0.0
Diarrhea	1	4.3	0	0.0	0	0.0	1	4.3
Rash	1	4.3	2	8.7	1	4.3	0	0.0
Paresthesia	1	4.3	0	0.0	0	0.0	0	0.0
Anemia	1	4.3	1	4.3	0	0.0	0	0.0
Increased aminotransferase	3	13.0	0	0.0	0	0.0	0	0.0
Increased creatinine	1	4.3	0	0.0	0	0.0	0	0.0
Hyperglycemia	1	4.3	0	0.0	0	0.0	1	4.3
Neutropenia	0	0.0	1	4.3	2	8.7	0	0.0
Febrile neutropenia	0	0.0	0	0.0	0	0.0	1	4.3

### Radiological and pathological response to neoadjuvant treatment

**Fig. 2:**
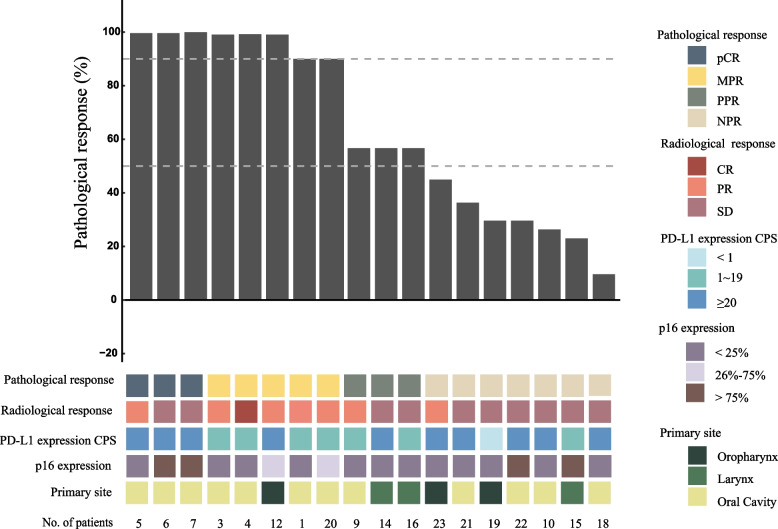
Waterfall plot of the characteristic of treatment response (*n* = 18). Each column indicates one patient, ranging from the highest to the lowest rate of pathological response. Corresponding sequence numbers of patients are labeled below. Two dashed horizontal lines denote 50% and 90% pathological responses. Three (16.7%) patients achieved pCR at the primary site, five (27.8%) achieved MPR (two among which were near-pCR, 1%), three (16.7%) achieved PPR, and seven (38.8%) were NPR

**Fig. 3:**
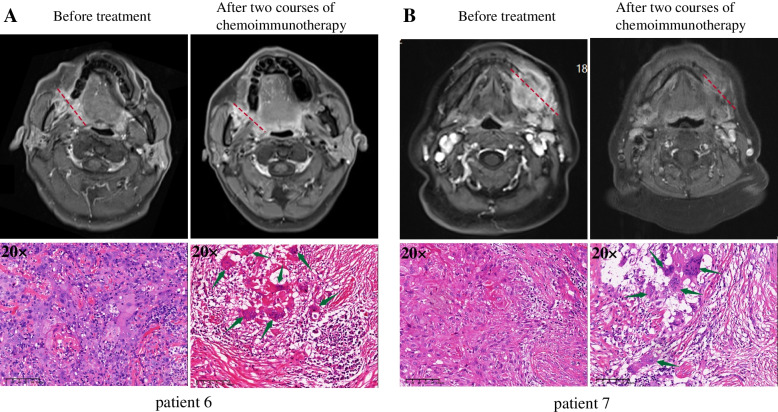
Magnetic resonance images (T1 enhanced sequence images) and H&E staining images (20 ×) of primary site tumor before and after two courses of chemo-immunotherapy. Patient 6(A) and patient 7(B), suffering the squamous cell carcinoma at the mandible (T4aN1M0) and gingiva (T2N3bM0) respectively. As for patient 6, the maximum diameter of tumor was about 23 mm before neoadjuvant treatment, which was significantly enhanced on enhanced scan. After neoadjuvant treatment, the maximum diameter of tumor was still about 23 mm, and the intensity of enhancement was not significantly reduced. For patient 7, before neoadjuvant treatment, the maximum diameter of tumor was about 32 mm, which was also significantly enhanced on enhanced scan. Though the maximum diameter of tumor shrank to about 25 mm after neoadjuvant treatment, and the intensity of enhancement was significantly reduced, the radiological response was still assessed to be SD. Surprisingly, both of them achieved pCR at the primary site after two courses of chemo-immunotherapy. Histopathological assays of pre-treatment show numerous infiltrating tumor nest with nuclear enlargement, nuclear hyperchromasia, prominent nucleoli and mitotic figures, which conforms to the characteristics of cancer cells.After treatment, histopathology revealed interstitial fibrous tissue hyperplasia, infiltration of multinucleated giant cells and chronic inflammatory cells (including lymphocyte and plasma cells infiltration), and no residual tumor inside the tumor bed. Histopathology revealed interstitial fibrous tissue hyperplasia, infiltration of multinucleated giant cells (arrows) and chronic inflammatory cells (including lymphocyte and plasma cells infiltration), and no residual tumor inside the tumor bed

Specific radiological and pathological responses to neoadjuvant treatment are shown in Fig. 2. The vast majority of patients (21/23, 91.3%) completed two cycles of induced treatment. Twenty patients were assessed for radiographic response to the combination of toripalimab and GP, according to RECIST 1.1. Among the 20 patients, 1 patinet (5.0%) achieved CR, 8 (40.0%) achieved PR, and 11 (55.0%) achieved SD. The ORR reached 45.0%. No progressive disease was observed. For two of the three patients who rejected surgery, the radiological evaluations were PR and SD, respectively (Supplemental Fig. 1A, B). Among patients who underwent radical surgery, the R0 resection rate was 100%. Three (16.7%) patients achieved pCR at the primary site, five (27.8%) achieved MPR (two of which were near-pCR, 1%), three (16.7%) achieved PPR, and seven (38.8%) achieved NPR (Fig. 2). Pathologically positive lymph nodes were found in 2 of 18 patients. The lymph nodes of 13 subjects were radiologically positive at baseline but pathologically negative after neoadjuvant immunohistochemistry. In addition, the radiological response was not compatible with pathology. As exemplified by patient 6 and 7, both patients achieved pCR by the pathological evaluation of the surgical specimen, although the evaluation of their radiological images was categorized as SD instead of PR or CR (Fig. 3A, B).

### Immunohistochemistry

**Fig. 4:**
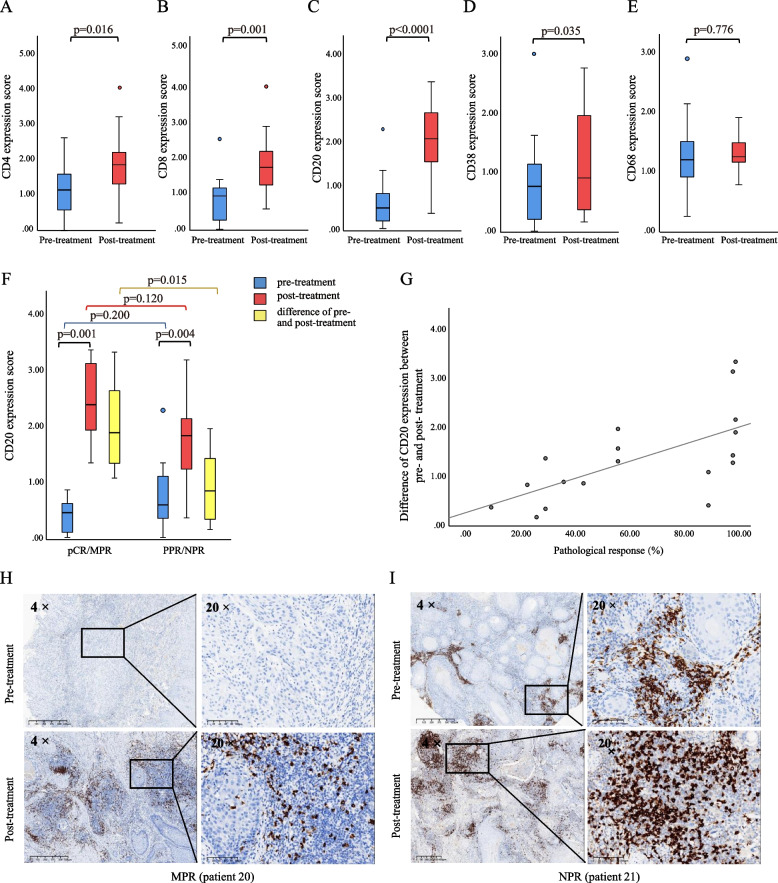
Box plots of immunohistochemical evaluation. **A**–**E** The expression of CD4, CD8, CD20, CD38, and CD68 in tissues pre- and post- treatment. **F** The difference in CD20 expression pre- and post-treatment in patients with pCR/MPR and PPR/NPR. **G** Spearman’s correlation analysis of the difference in CD20 expression pre- and post-treatment with pathological response rates (*p* < 0.05, *r* = 0.75). (H–I) CD20 expression pre- and post-treatment in the patient with MPR (**G**) and NPR (**I**)

To explore the potential association between the pathologic response and immune biomarkers, we performed immunohistochemistry on available paired pre-treatment biopsies and surgical resection specimens from 18 patients. There was a significant increase in the expression of CD4 (*p* = 0.016), CD8 (*p* = 0.001), CD20 (*p* < 0.0001), and CD38 (*p* = 0.035) in tumors after neoadjuvant chemotherapy (paired test), whereas no significant difference was found in the expression of CD68 (Fig. 4A–E). There were no significant differences in the expression of CD4, CD8, CD20, and CD38 pre- and post-treatment between patients with pCR/MPR and PPR/NPR (Supplemental Fig. 2). However, the increase in CD20 expression after neoadjuvant treatment in patients with pCR/MPR was significantly higher than that in patients with PPR/NPR (*p* = 0.015) (Fig. 4F). The increase in CD20 expression after neoadjuvant treatment positively correlated with the pathological response rate (Fig. 4G). The correlation coefficient (by Spearman’s correlation test) between the two variables was 0.75 (*p* < 0.0001). Immunohistochemical images of CD20 expression pre- and post-treatment in patients with MPR and NPR are shown in Fig. 4H and I. In addition, we analyzed the peripheral T lymphocyte counts, including CD3, CD4, and CD8 lymphocytes, pre- and post-treatment, but no significant difference was observed between the pCR/MPR and PPR/NPR groups (Supplemental Fig. 3). The mean percentage of PD-L1 positive cells in the tumor bed pre- and post-treatment was 38.6 and 48.8%, respectively, although the difference was not statistically significant. There was no significant difference in the percentage of PD-L1 positive cells in the tumor bed between the pCR/MPR and PPR/NPR groups (Supplemental Fig. 4).

### Follow-up

**Fig. 5:**
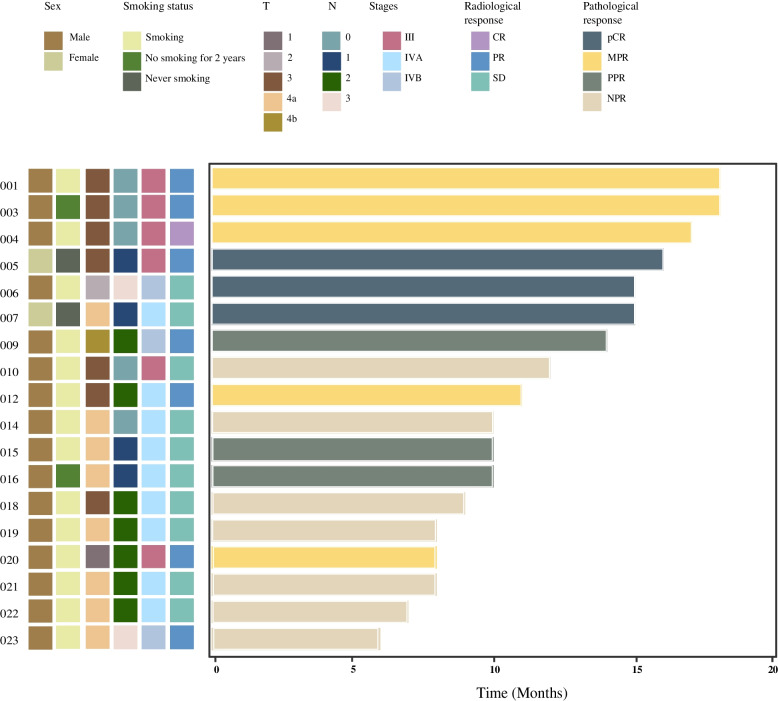
Swimming plot of survival. Each lane indicates one patient, ranked by the survival time. Corresponding sequence numbers of patients are labeled on the left. Characteristics of patients are shown on the top. No disease progression and death happened until writing. The median follow-up from the first day of treatment was 10.5 months, ranging from 6 to 18 months

By July 2022, no case of disease progression had occurred, and the median follow-up from the first day of treatment was 10.5 months, ranging from 6 to 18 months (Fig. 5). The data for 1-year and 3-year OS rates and DFS rates will be shown in subsequent publications.

## Discussion

Collectively, the neoadjuvant combination therapy of toripalimab plus GP was proven safe and feasible for resectable locally advanced HNSCC. No disease progression was recorded in this study, and the addition of PD-1 inhibitor to neoadjuvant chemotherapy, in contrast to neoadjuvant chemotherapy or immunotherapy alone, did not give rise to more risks to the patients. The adverse effects spectrum we presented was similar to other clinical studies reported on neoadjuvant GP chemotherapy or immunotherapy alone in HNSCC [22, 23]. Regarding neoadjuvant chemoimmunotherapy, the rate of grade 3/4 TRAEs in our study was 21.7%, lower than that reported by Zinner et al. (37%) [24]. TRAEs, such as neutropenia or febrile neutropenia, might be more likely to be caused by chemotherapy. In addition, we observed a seemingly higher incidence of rashes after combined immunotherapy than that after chemotherapy alone, perhaps due to the superimposition of adverse effects caused by the two.

The data on the pathological response rates of immunotherapy alone varied significantly, with the evaluation indices differing. Razan et al. reported that the pCR rate ranged from 0 to 16.7%, and the mean MPR rate was 9.7% in resectable HNSCC treated with immunotherapy alone [23]. In our study, 27.8% of the patients achieved MPR at the primary site after neoadjuvant chemotherapy, significantly higher than the MPR rate reported previously in patients with neoadjuvant immunotherapy alone. Studies of neoadjuvant chemoimmunotherapy in locally advanced HNSCC are rare. Zinner et al. reported a pCR rate of 42% in locally advanced HNSCC treated with neoadjuvant nivolumab plus carboplatin and paclitaxel; however, the final results have not yet been published [24]. The different characteristics of the included patients may contribute to the varied results of the endpoints. The exact efficacy of neoadjuvant chemotherapy combined with chemotherapy in HNSCC requires confirmation in larger clinical studies. Kunyu Yang et al. recently published the results of a phase 2 trial involving patients with resectable stage III-IVB HNSCC who received chemotherapy [albumin-bound paclitaxel (or docetaxel) plus cisplatin] and camrelizumab for three cycles. In their study, 27 patients underwent surgery, with an R0 resection rate of 92.6% [25]. The pCR rate was 37.0%, and the MPR rate was 74.1%, with tolerable safety profile. In contrast, their study reported higher pCR and MPR rates than our study, however, they administered three cycles of neoadjuvant chemoimmunotherapy, while ours only had two cycles. Up to now, two cycles of neoadjuvant drug therapy have always been preferred for the population with resectable carcinoma. Many typical researched has confirmed the safety and applicability of two cycles neoadjuvant treatment [26–28]. In the mode, the planned surgery would not be significantly affected even once the neoadjuvant therapy helped little. In addition, the time window between the end of neoadjuvant therapy and surgery was different between the two studies, which may have contributed to the different results. Nevertheless, the R0 resection rate was higher in our study, which was strongly related to the intensity of subsequent adjuvant therapy and the prognosis of patients. Both studies are phase I-II studies with small samples, and the exact efficacy of neoadjuvant chemoimmunotherapy needs to be confirmed by larger phase III studies. More importantly, the optimal regimen and mode of neoadjuvant therapy still needs to be further explored.

Additionally, we found that the radiological response did not coincide with the pathological response. As exemplified by patients No. 06 and 07, they achieved a complete pathological response but were evaluated as SD according to radiological images per Recist 1.1. Considering that immunotherapy can induce infiltration of immune cells within the tumor, the lesions observed on imaging after neoadjuvant therapy in these two patients may reflect an immune response because no tumor cell survival was confirmed by pathology. This phenomenon may be caused by a large number of immune cells infiltrating the lesion, or it might be caused by the accumulation of microvascular rupture, cell effusion, and necrotic tumor cells [29]. The pseudo-progression rate of HNSCC after immunotherapy is reported to be 1.79% [30]. This indicates that imaging cannot replace pathology in evaluating the efficacy of immunotherapy and that an SD evaluated by imaging might be a pseudo-SD. In contrast, the efficacy of neoadjuvant therapy in some patients was PR in imaging evaluation but PPR/NPR in pathological evaluation. This suggests that the immune cell infiltration in tumors of those patients might be relatively less, and the PR of those patients might be more dependent on the role of chemotherapy due to the heterogeneity of the tumor. Comparison from the aspect of radiomics might be a better match to a pathological assessment. However, our data related to radiomics are incomplete. Radiomic comparison before and after neoadjuvant treatment cannot be evaluated. That would be a very interesting direction for further research.

In our study, the expression of CD20 in tumors significantly increased after neoadjuvant therapy, and the increase in CD20 expression in patients with pCR/MPR was significantly higher than that in patients with PPR/NPR after neoadjuvant therapy. Correlation analysis confirmed that the increase in CD20 expression after neoadjuvant therapy was correlated with the pathological response rate. Those results suggested that B cells might also play an important role in antitumor immunity, in addition to T cells. Previous studies have shown that B cells may enhance the response of T cells by producing antibodies against tumor antigens, stimulating the secretion of cytokines and chemokines, and acting as local antigen presenting cells etc. to maintain long-term immunity [31]. We would conduct further research on the synergistic effect of B cells and T cells, and the relationship between B cells and immune response to PD-1 inhibitors and patient prognosis after neoadjuvant chemoimmunotherapy in HNSCC in the future. Previous studies have demonstrated that more CD20 + immune cells in tumors after neoadjuvant therapy are associated with better outcomes in HNSCC or breast cancer [32, 33], supported by our research to some extent. According to previous literature [34, 35], CD38 expression is associated with M1 macrophages, which produce pro-inflammatory cytokines and exert anti-tumor effects. Coexpression of CD38 and CD68 on macrophages is correlated with improved prognosis by increasing the M1 to M2 macrophage ratio. Our results presented that although CD68 expression was not significantly increased after neoadjuvant therapy, CD38 expression was elevated after neoadjuvant therapy with statistically significance. That also gave us a hit that neoadjuvant chemoimmunotherapy might promote M1 polarization in the tumor microenvironment.

PD-L1 expression in tumor cells and associated immune cells predicts better survival for immunotherapy in HNSCC [6]. The relationship between PD-L1 expression and the pathological response rate has also been explored. PD-L1 positive tumor cells were reported to be strongly correlated with pCR in breast cancer [33]. Baseline PD-L1 expression in tumor cells or immune cells was also a predictor of pCR or pTR ≥ 10% in HNSCC [8, 36]. However, Vos et al. showed no difference in baseline PD-L1 expression between patients with pCR/MPR and those with NPR in HNSCC [37]. In our study, although there was no statistical difference between the baseline PD-L1 CPS score in the tumor bed and the pathological response rate, we observed that the PD-L1 CPS scores of all 3 patients with pCR were 100. This suggests that high PD-L1 expression in tumors might be a huge advantage for patients to achieve pCR. However, higher expression of PD-L1 did not guarantee better efficacy of immunotherapy due to the influence of other links in immune activation and response processes.

The current study was limited by the small sample size. In addition, the efficacy of neoadjuvant immunotherapy combined with chemotherapy in HNSCC needs to be verified by more large-scale clinical studies, and large-scale controlled studies should be conducted in the future to explore the advantages of immunotherapy combined with chemotherapy over chemotherapy alone. We will continue to report the survival time and its correlation with the pathological response rates. In addition, some patients were relatively insensitive to immunotherapy and chemotherapy. In the future, we will analyze the mechanism of differential responses in patients through genetic testing, screen sensitive populations, and try to increase the response of patients insensitive to chemoimmunotherapy. Our new clinical trial will add low-dose radiotherapy to chemoimmunotherapy to improve response to chemoimmunotherapy (NeoRTPC02).

## Conclusions

The trial demonstrated favorable toxicity and improved efficacy of neoadjuvant gemcitabine and cisplatin combined with toripalimab in patients with HNSCC. CD20 is a potential biomarker of pathological responses to chemoimmunotherapy. This combination should be encouraged in larger clinical trials.

## Supplementary Information

**Additional file 1. **

**Additional file 2. **

**Additional file 3. **

**Additional file 4. **

## Data Availability

The datasets used and/or analysed during the current study are available from the corresponding author on reasonable request.
