# Supplementary figures and images for: Neoadjuvant toripalimab combined with gemcitabine and cisplatin in resectable locally advanced head and neck squamous cell carcinoma (NeoTGP01): An open label, single-arm, phase Ib clinical trial
Source: J Exp Clin Cancer Res. 2022 Oct 12;41:300. doi: 10.1186/s13046-022-02510-2 (PMC9558942; doi:10.1186/s13046-022-02510-2)

Supplement figure 1

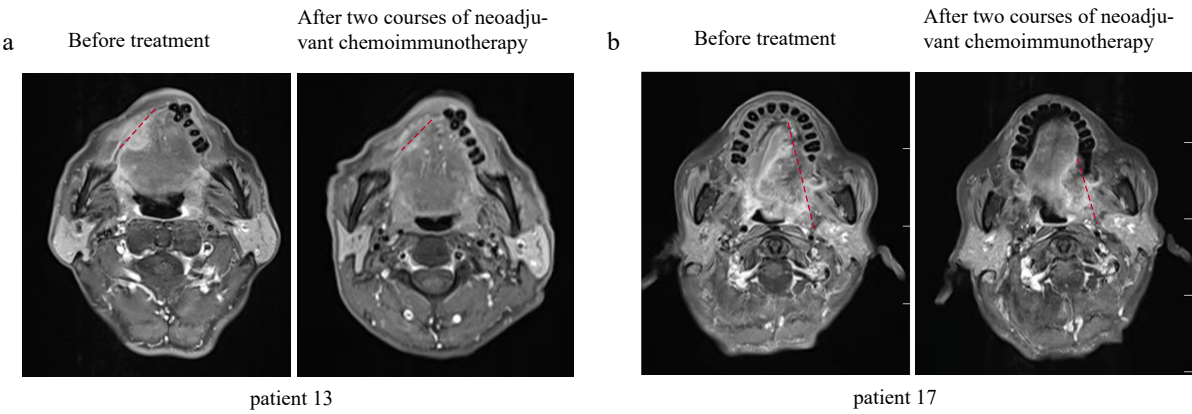

Supplement: Supplementary file 1 — Additional file 1. [file 13046_2022_2510_MOESM1_ESM.pdf]

Supplement figure 2

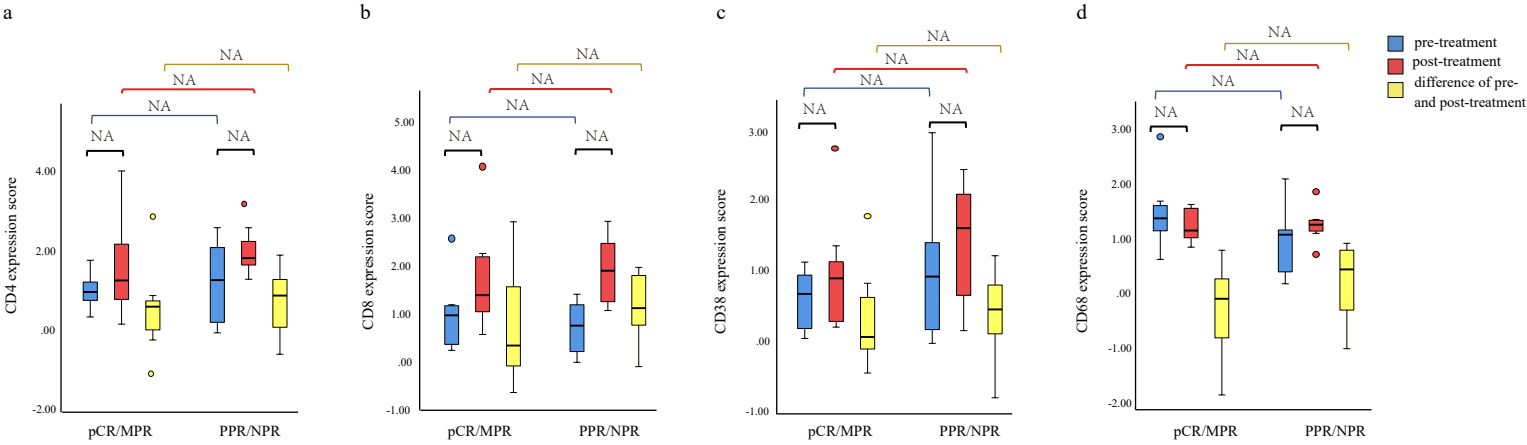

Supplement: Supplementary file 2 — Additional file 2. [file 13046_2022_2510_MOESM2_ESM.pdf]

Supplement figure 3

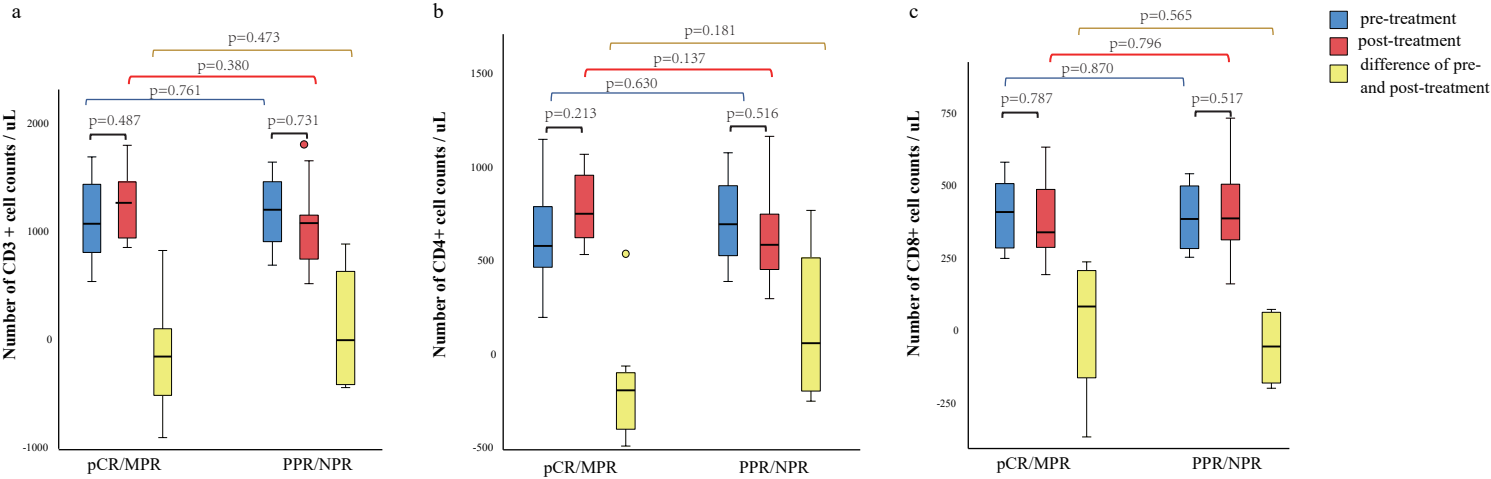

Supplement: Supplementary file 3 — Additional file 3. [file 13046_2022_2510_MOESM3_ESM.pdf]

Supplement figure 4

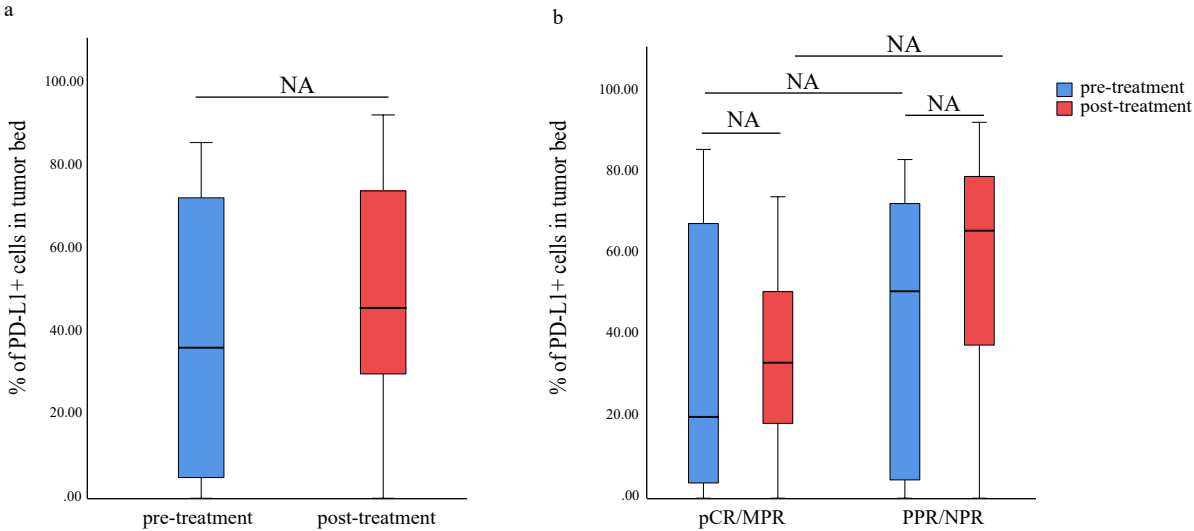

Supplement: Supplementary file 4 — Additional file 4. [file 13046_2022_2510_MOESM4_ESM.pdf]
